# Atypical Presentation and Successful Management of Combined Hyperthyroidism and Adrenal Insufficiency in a Young Male

**DOI:** 10.7759/cureus.66150

**Published:** 2024-08-04

**Authors:** Zeyad Khalil, Reham E Hussein, Eman Z Al-Abbedien

**Affiliations:** 1 College of Medicine, October 6th university, Cairo, EGY; 2 College of Medicine, October 6th University, Cairo, EGY; 3 Geriatrics, Bin-Seuif Hospital, Beni Suef, EGY

**Keywords:** antithyroid medications, glucocorticoid replacement therapy, multidisciplinary approach, hormonal assays, endocrine disorders, adrenal insufficiency, hyperthyroidism

## Abstract

This case report details the unusual presentation and successful management of a 25-year-old male diagnosed with both hyperthyroidism and adrenal insufficiency. The patient initially presented with symptoms of fatigue, weight loss, and palpitations, with no significant past medical history. Further evaluation revealed elevated thyroid hormone levels and decreased cortisol levels, confirming the diagnosis of concurrent hyperthyroidism and adrenal insufficiency. The complexity of managing these coexisting endocrine disorders required a multidisciplinary approach. Techniques utilized included detailed hormonal assays, imaging studies, and dynamic endocrine testing. The therapeutic regimen involved the administration of antithyroid medications, beta-blockers for symptom control, and glucocorticoid replacement therapy. This report underscores the importance of considering multiple endocrine disorders in patients with nonspecific systemic symptoms and highlights the need for individualized treatment plans to address the unique challenges presented by such comorbidities.

## Introduction

Hyperthyroidism is a pathological condition characterized by the excessive synthesis and secretion of thyroid hormones (thyroxine (T4) and triiodothyronine (T3)) by the thyroid gland, resulting in a hypermetabolic state [1]. The thyroid gland, a key endocrine organ located in the anterior neck, is integral to metabolic regulation and exerts widespread effects on various physiological processes through the actions of T4 and T3 [1]. These hormones are crucial in modulating basal metabolic rate, thermogenesis, and the sympathetic nervous system’s activity and influencing cardiovascular, gastrointestinal, and neuromuscular functions [1,2]. The etiology of hyperthyroidism is multifactorial, with Graves’ disease being the predominant cause, accounting for approximately 60-80% of cases [1-2]. Graves’ disease is an autoimmune disorder wherein thyroid-stimulating immunoglobulins (TSI) bind to and activate the thyrotropin (thyroid-stimulating hormone (TSH)) receptor, causing unregulated thyroid hormone production and diffuse thyroid enlargement (goiter). The pathophysiology involves a complex interplay of genetic susceptibility and environmental triggers, leading to the loss of immune tolerance and the production of autoantibodies [1-4]. Other etiologies include toxic multinodular goiter and toxic adenoma, where autonomously functioning thyroid nodules hypersecrete thyroid hormones independent of TSH control, often due to somatic mutations in the TSH receptor or G-protein signaling pathways [5].

Biochemically, hyperthyroidism results in elevated serum free T4 and T3 levels, with concomitant suppression of TSH due to negative feedback inhibition at the hypothalamic-pituitary axis [6]. The excess thyroid hormones enhance metabolic processes, leading to increased oxygen consumption and heat production [6]. Clinically, this manifests as weight loss despite increased appetite, heat intolerance, hyperhidrosis, tremors, palpitations, and anxiety. The cardiovascular system is particularly affected, with increased heart rate, contractility, and cardiac output, predisposing patients to arrhythmias such as atrial fibrillation [6,7]. Neuromuscular symptoms include hyperreflexia and proximal muscle weakness due to the catabolic effects of thyroid hormones on muscle proteins [7]. Diagnosis of hyperthyroidism is primarily biochemical, involving the measurement of serum TSH (typically suppressed) and elevated serum free T4 and T3 levels. Thyroid autoantibodies, including TSI and thyroid peroxidase antibodies (TPO), aid in confirming the autoimmune etiology. Imaging studies, such as thyroid ultrasound and radioactive iodine uptake scans, are utilized to differentiate between diffuse and nodular thyroid disease and to assess functional activity [8].

Conversely, adrenal insufficiency represents a distinct yet equally critical endocrine disorder characterized by inadequate production of glucocorticoids, and sometimes mineralocorticoids, by the adrenal cortex [8]. The adrenal glands, located atop the kidneys, are critical in stress response, metabolism, immune function, and electrolyte balance through the secretion of cortisol and aldosterone [8]. Adrenal insufficiency can be categorized as primary, secondary, or tertiary. Primary adrenal insufficiency, or Addison’s disease, often results from autoimmune destruction of the adrenal cortex but can also be caused by infections (e.g., tuberculosis, HIV), adrenal hemorrhage, or metastatic disease [9]. Secondary adrenal insufficiency arises from inadequate adrenocorticotropic hormone (ACTH) production due to pituitary pathology, whereas tertiary adrenal insufficiency stems from hypothalamic dysfunction leading to insufficient corticotropin-releasing hormone (CRH) secretion [9]. The pathophysiology of adrenal insufficiency involves disruption of the hypothalamic-pituitary-adrenal (HPA) axis, resulting in deficient cortisol production. Cortisol is vital for gluconeogenesis, immune modulation, and maintaining vascular tone. Its deficiency leads to a constellation of symptoms including profound fatigue, anorexia, weight loss, hypotension, and electrolyte imbalances such as hyponatremia and hyperkalemia. In primary adrenal insufficiency, hyperpigmentation of the skin occurs due to elevated ACTH levels stimulating melanocortin receptors [10].

Biochemical diagnosis of adrenal insufficiency involves measuring early morning serum cortisol and ACTH levels. Low cortisol levels with elevated ACTH indicate primary adrenal insufficiency, whereas low or inappropriately normal ACTH levels suggest secondary or tertiary causes. The ACTH stimulation test further evaluates adrenal function by measuring cortisol response to exogenous ACTH administration [10,11]. Management of hyperthyroidism includes antithyroid medications (methimazole, propylthiouracil), beta-blockers for symptomatic relief, and definitive treatments such as radioactive iodine ablation or thyroidectomy for refractory cases. In contrast, adrenal insufficiency treatment requires glucocorticoid replacement (hydrocortisone, prednisone) and mineralocorticoid replacement (fludrocortisone) in primary insufficiency. The coexistence of these two endocrine disorders presents a unique clinical challenge, necessitating a comprehensive and individualized therapeutic approach to address the complex interplay of hormonal imbalances [11-13].

## Case presentation

A 25-year-old male presented to the emergency department with complaints of severe fatigue, unintentional weight loss of 10 kg over the past two months, and frequent palpitations. The patient, accompanied by his mother, also reported increased sweating, anxiety, and intermittent dizziness. He had visited the hospital three times over the past week due to the progressive worsening of these symptoms, initially presenting alone and later accompanied by his family members. The patient had no significant past medical history, no history of recent infections, and no use of medications or illicit substances. His family history was unremarkable for endocrine disorders. The patient was a non-smoker, consumed alcohol occasionally, and worked as a software engineer. He led a moderately active lifestyle, engaging in regular physical exercise. On physical examination, the patient appeared anxious and agitated, with a tremor in the hands. His vital signs were notable for a heart rate of 110 beats per minute, blood pressure of 130/80 mmHg, and a temperature of 37.8°C. Neurological examination revealed hyperreflexia and proximal muscle weakness. The thyroid gland was diffusely enlarged and non-tender on palpation. Given the presentation of weight loss, palpitations, and tremors, initial differential diagnoses included hyperthyroidism, pheochromocytoma, and anxiety disorder. The patient was admitted for further evaluation and management.

Laboratory tests were conducted as shown in Table 1, revealing a complete blood count (CBC) within normal limits and a comprehensive metabolic panel showing elevated liver enzymes. Thyroid function tests indicated suppressed TSH levels (<0.01 µIU/mL) and elevated free T4 (3.2 ng/dL) and T3 (325 ng/dL), confirming hyperthyroidism. Additionally, cortisol levels were measured due to the patient’s significant fatigue and weight loss, revealing a morning cortisol level of 2.5 µg/dL, which was suggestive of adrenal insufficiency. This prompted an ACTH stimulation test, which showed a suboptimal increase in cortisol levels (baseline 2.5 µg/dL, 30-minute post-ACTH 5.0 µg/dL), confirming the diagnosis of adrenal insufficiency. Imaging studies included a thyroid ultrasound, which showed diffuse thyroid enlargement with increased vascularity consistent with Graves’ disease which was confirmed with a radioactive uptake iodine test (RAIU). An abdominal CT scan was performed to assess the adrenal glands, revealing bilaterally atrophic adrenal glands, further supporting the diagnosis of primary adrenal insufficiency (Addison’s disease).

**Table 1:** Laboratory results ACTH: adrenocorticotropic hormone

Test	Result	Reference Range/Normal Values	Units
White blood cells (WBC)	7.5	4.0-11.0	x10^3^/µL
Hemoglobin (Hgb)	14.5	13.5-17.5	g/dL
Platelets	250	150-450	x10^3^/µL
Alanine aminotransferase (ALT)	75	7-56	U/L
Aspartate aminotransferase (AST)	60	10-40	U/L
Thyroid-stimulating hormone (TSH)	<0.01	0.4-4.0	µIU/mL
Free thyroxine (Free T4)	3.2	0.8-1.8	ng/dL
Free triiodothyronine (Free T3)	325	80-200	ng/dL
Morning cortisol	2.5	6-23	µg/dL
Post-ACTH cortisol (30 min)	5.0	>18	µg/dL
ACTH	67	10-60	pg/mL

**Figure 1:**
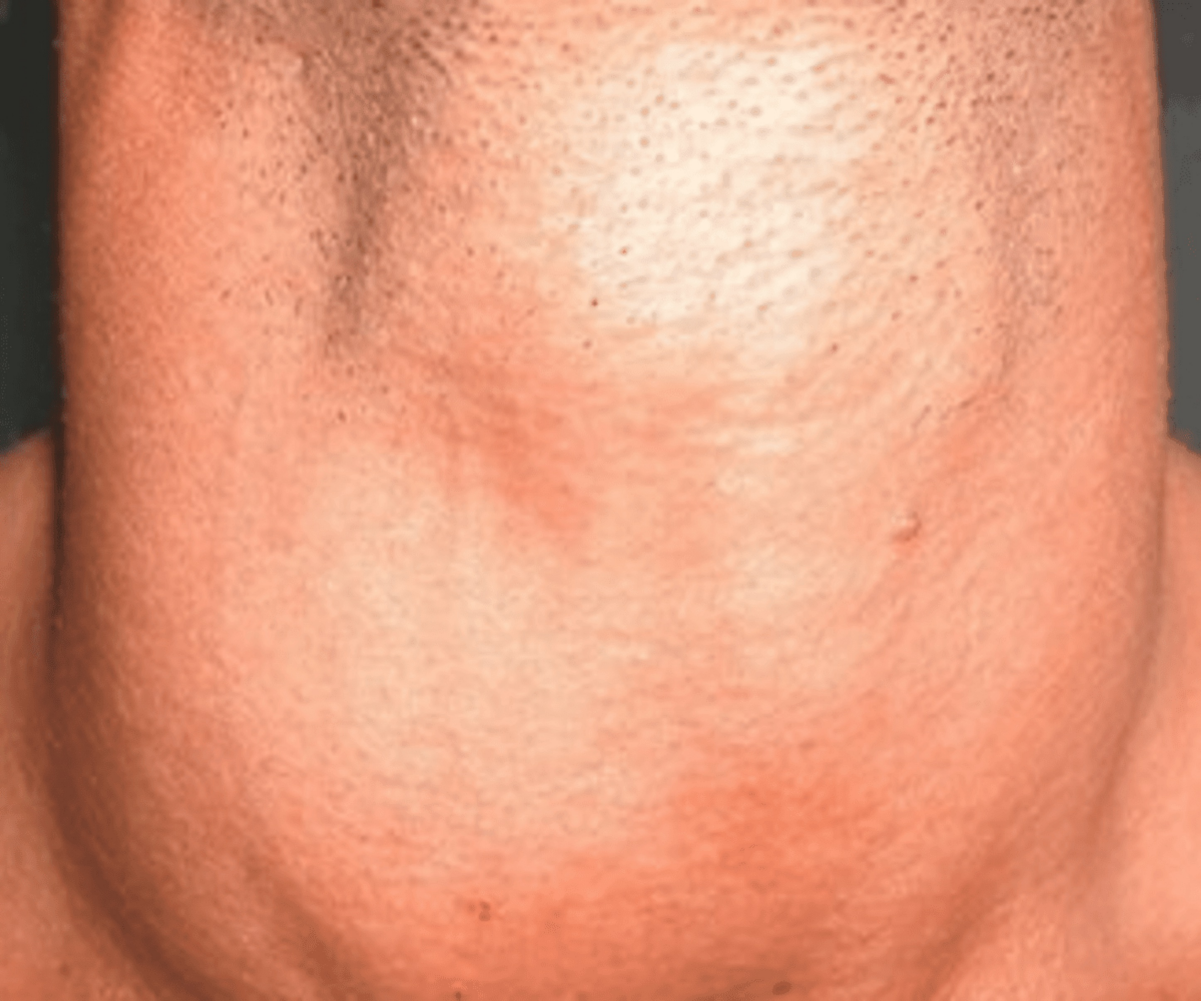
Diffuse Smooth Swelling of the Thyroid Gland

Figure 1 shows the thyroid gland of the patient described in the case report. The image depicts a diffuse, smooth swelling of the thyroid gland, indicative of a goiter. This goiter is characterized by a symmetrical and uniform enlargement of the thyroid tissue, which is a common finding in conditions such as Graves’ disease. In this patient, the thyroid gland is prominently enlarged and non-tender on palpation, as observed during the physical examination. The diffuse nature of the swelling aligns with the typical presentation of diffuse thyroid hyperplasia seen in Graves’ disease, where the entire thyroid gland is uniformly enlarged due to autoimmune stimulation of the TSH receptor.

**Figure 2:**
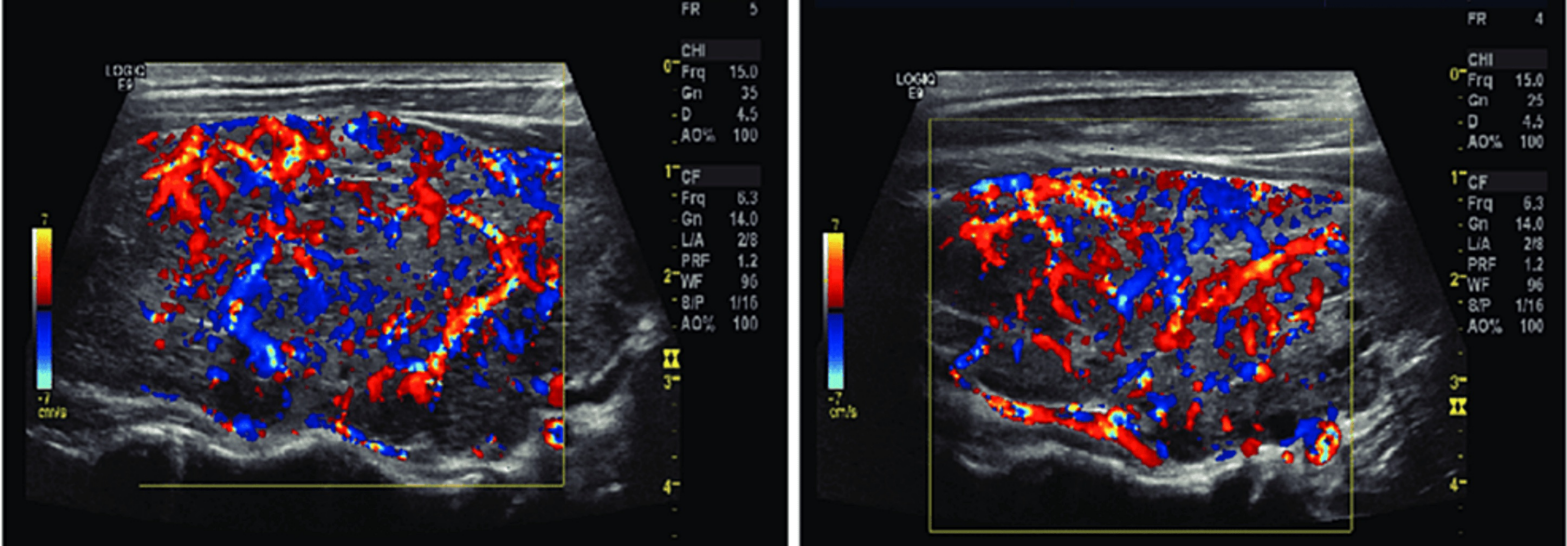
Doppler Ultrasonography Longitudinal Views of the Thyroid Gland Showing Diffuse Enlargement and Increased Vascularity

**Figure 3:**
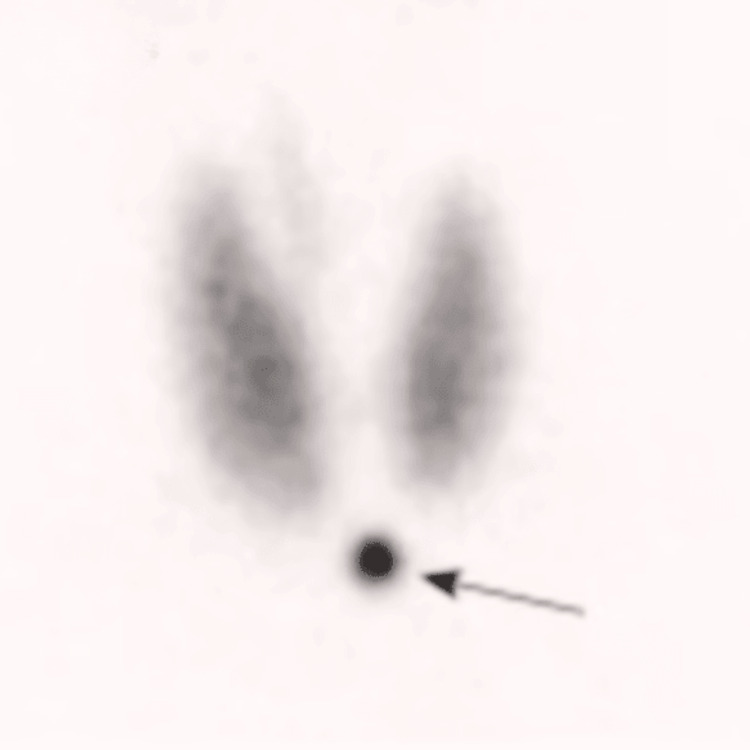
Radioactive Iodine Uptake Test Confirming Suspicion of Graves’ Disease

Doppler ultrasonography longitudinal views of the right (A) and left (B) lobes of the thyroid gland are shown in Figure 2, illustrating features consistent with Graves’ disease. These images, obtained using Doppler ultrasonography, display longitudinal (sagittal) views, meaning the ultrasound transducer was aligned parallel to the length of the thyroid lobes, providing a vertical section through the gland. Color Doppler imaging is used to visualize blood flow within the thyroid tissue, with red indicating blood flow toward the transducer and blue indicating blood flow away from the transducer. Both images show diffuse enlargement of the thyroid gland, characteristic of Graves’ disease, and extensive color signals throughout both lobes, indicative of increased vascularity. This hypervascular pattern reflects the increased blood flow due to hyperfunctioning thyroid tissue, typical in Graves’ disease. Additionally, the thyroid parenchyma appears relatively homogeneous without nodules, consistent with diffuse thyroid disease rather than nodular thyroid disease. These images collectively represent a thyroid gland affected by Graves’ disease, showing both structural enlargement and significantly increased blood flow, hallmarks of this condition. To confirm the presence of Graves’ disease, a radioactive iodine uptake test (RAIU) was conducted. The results showed diffuse and symmetrical thyroid uptake, along with reduced salivary gland uptake as shown in Figure 3.

**Figure 4:**
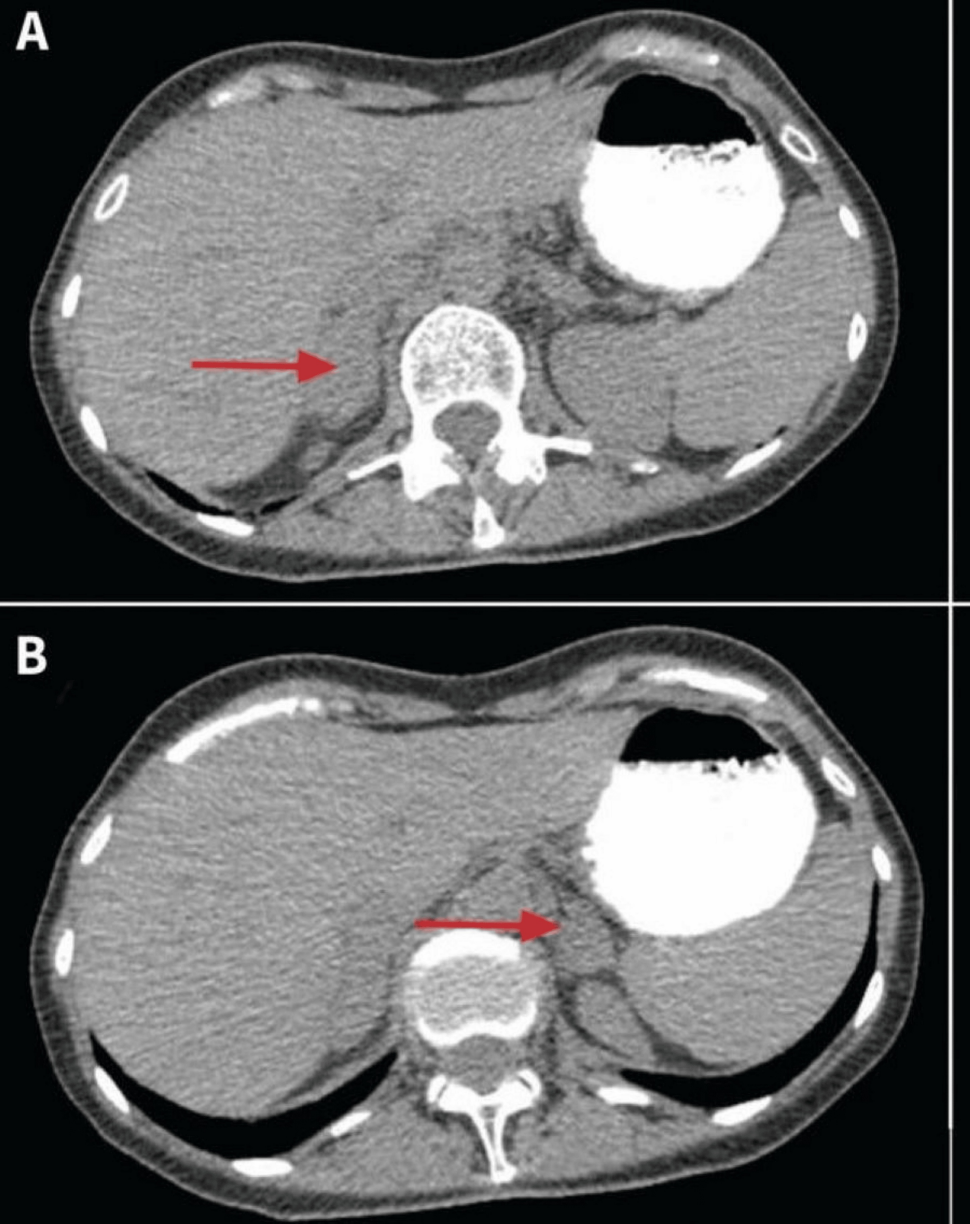
Abdominal CT Scan Showing Bilaterally Atrophic Adrenal Glands Pretreatment

For confirmation of adrenal insufficiency suspicion, a cross-sectional abdominal CT scan was conducted (Figure 4) showing bilaterally atrophic adrenal glands. The arrows point to the adrenal glands, which appear significantly smaller than normal. This imaging finding is consistent with primary adrenal insufficiency, also known as Addison’s disease. In primary adrenal insufficiency, the adrenal glands are often atrophic due to autoimmune destruction, which is the most common cause, but other causes can include infections, hemorrhage, or metastatic disease. The atrophy is typically bilateral, affecting both adrenal glands, as seen in the image. The CT scan reveals decreased size and volume of the adrenal glands, which is indicative of chronic damage and loss of functional adrenal tissue. This loss leads to insufficient production of essential hormones such as cortisol and aldosterone, resulting in the clinical manifestations of Addison’s disease. The diagnosis is supported by clinical presentation, biochemical tests showing low cortisol and high ACTH levels, and imaging findings like those depicted here.

Given the dual diagnosis, the management plan required careful consideration to avoid precipitating an adrenal crisis. The patient was started on methimazole 10 mg twice daily to manage hyperthyroidism and propranolol 40 mg three times daily to control sympathetic symptoms. For adrenal insufficiency, hydrocortisone replacement therapy was initiated at 20 mg in the morning and 10 mg in the evening, along with fludrocortisone 0.1 mg daily to address mineralocorticoid deficiency. Over the course of hospitalization, the patient’s symptoms gradually improved. His heart rate normalized, and he reported decreased palpitations and anxiety. Follow-up thyroid function tests showed a reduction in T4 and T3 levels, and cortisol levels stabilized with hydrocortisone therapy. The patient was educated on the importance of medication adherence, signs of adrenal crisis, and the need for regular follow-up with endocrinology.

## Discussion

This case report presents the unique and complex diagnostic challenge of a young male with concurrent hyperthyroidism and primary adrenal insufficiency (Addison’s disease). The coexistence of these two endocrine disorders is rare and poses significant diagnostic and therapeutic challenges. The patient’s presentation with overlapping symptoms of fatigue, weight loss, and palpitations initially complicated the differential diagnosis, necessitating a comprehensive and multidisciplinary approach to identify and manage the underlying conditions accurately.

The diagnosis of hyperthyroidism was confirmed through a combination of clinical assessment and advanced diagnostic techniques. The use of thyroid function tests, specifically measuring suppressed TSH and elevated serum free T4 and T3 levels, was crucial in establishing hyperthyroidism. These biochemical findings were essential in differentiating hyperthyroidism from other potential causes of the patient’s symptoms, such as anxiety disorders or pheochromocytoma [1,3]. The thyroid ultrasound provided further confirmation, revealing diffuse enlargement and increased vascularity characteristic of Graves’ disease, thereby reinforcing the clinical diagnosis [2,9].

In parallel, the identification of primary adrenal insufficiency added another layer of complexity to this case. The initial suspicion of adrenal insufficiency arose from the patient’s profound fatigue and significant weight loss, which were not fully explained by hyperthyroidism alone. The measurement of morning cortisol levels revealed significantly low cortisol, prompting an ACTH stimulation test. The suboptimal increase in cortisol levels post-ACTH administration confirmed primary adrenal insufficiency. This biochemical diagnosis was further corroborated by imaging studies; the abdominal CT scan showed bilaterally atrophic adrenal glands, a hallmark of Addison’s disease, thus providing a definitive anatomical correlate to the biochemical findings [4,11,12].

The therapeutic management of this patient required careful coordination to address both endocrine disorders simultaneously without precipitating an adrenal crisis. The administration of methimazole effectively reduced thyroid hormone synthesis, alleviating hyperthyroid symptoms [7,10]. Propranolol was used to manage sympathetic overactivity, providing symptomatic relief from palpitations and anxiety [8]. Concurrently, hydrocortisone and fludrocortisone replacement therapies were initiated to treat adrenal insufficiency.

The dosages and medications prescribed for the patient were selected based on established clinical guidelines and standards of care for managing hyperthyroidism and primary adrenal insufficiency. For hyperthyroidism, methimazole and propranolol were used to control thyroid hormone levels and sympathetic symptoms, respectively. In managing adrenal insufficiency, hydrocortisone and fludrocortisone were prescribed according to recommended replacement therapy protocols. The hydrocortisone dosage was adjusted to prevent an adrenal crisis, and follow-up assessments were made to ensure appropriate dosing. Plasma renin and aldosterone levels were already measured for the patient through his personal doctor who evaluated the presence of mineralocorticoid deficiency, confirming the need for fludrocortisone therapy. This comprehensive approach ensured adherence to clinical standards and effective management of the patient’s conditions. The precise dosing and titration of these medications were critical to ensure adequate glucocorticoid and mineralocorticoid replacement while avoiding potential complications such as adrenal crisis or exacerbation of hyperthyroid symptoms [5,6,13].

This case underscores the importance of a thorough and systematic approach in the diagnosis and management of patients with complex presentations involving multiple endocrine disorders. The use of detailed hormonal assays, imaging studies, and dynamic endocrine testing were instrumental in elucidating the underlying pathologies. Moreover, the individualized treatment plan, involving a combination of antithyroid drugs, beta-blockers, and steroid replacement, highlights the necessity of a tailored therapeutic approach in managing such intricate cases. This report contributes to the existing literature by illustrating the challenges and strategies in diagnosing and treating coexisting hyperthyroidism and primary adrenal insufficiency, emphasizing the need for vigilance and a multidisciplinary approach in similar clinical scenarios [3-6,10-13].

## Conclusions

To differentiate between hyperthyroidism and primary adrenal insufficiency, diagnostic steps included laboratory tests showing suppressed TSH and elevated free T4 and T3 (confirming hyperthyroidism), low morning cortisol levels, and a suboptimal ACTH stimulation test response (indicating adrenal insufficiency). Imaging revealed diffuse thyroid enlargement consistent with Graves’ disease and bilaterally atrophic adrenal glands. These methods were essential for accurate diagnosis and treatment. The patient was advised on medication adherence, adrenal crisis signs, stress management, and regular follow-up to manage both conditions effectively.
